# Different stages of the infection cycle are enriched for *Campylobacter* strains with distinct phenotypes and levels of fluoroquinolone resistance

**DOI:** 10.1099/mic.0.001349

**Published:** 2023-06-23

**Authors:** Gillian Carney, Bart C. Weimer, Marguerite Clyne, Tadhg Ó Cróinín

**Affiliations:** ^1^​ School of Biomolecular and Biomedical Science, University College Dublin, Belfield, Dublin 4, Ireland; ^2^​ School of Veterinary Medicine, Population Health and Reproduction, 100K Pathogen Genome Project, UC Davis, Davis, California, USA; ^3^​ School of Medicine, University College Dublin, Belfield, Dublin 4, Ireland

**Keywords:** campylobacter, fluoroquinolone resistance, virulence

## Abstract

*
Campylobacter* species are the leading cause of bacterial diarrhoea worldwide and consumption of contaminated chicken meat is the most common route of infection. Chickens can be infected with multiple strains of *Campylobacter* and during the infection cycle this pathogen must survive a wide variety of environments. Numerous studies have reported a high degree of genetic variability in this pathogen that can use antigenic and phase variation to alter the expression of key phenotypes. In this study the phenotypic profile of isolates from freshly slaughtered chickens, chicken products in the supermarket and stool samples from infected patients were compared to identify phenotypic changes during the passage of the bacteria through the infection cycle. Isolates from different stages of the infection cycle had altered phenotypic profiles with isolates from human stool samples showing a lower ability to form a biofilm and an increased ability to associate with epithelial cells *in vitro*. Resistance to fluoroquinolones was found in all cohorts but at a much higher occurrence (94%) in isolates from supermarket chicken. Isolates displaying high levels of resistance to fluoroquinolones also were more likely to display a higher tolerance to growth in the presence of oxygen. In conclusion, isolates with specific phenotypes appear to be overly represented at different stages of the infection cycle suggesting that environmental stresses may be enriched for strains with these phenotypes.

## Introduction

*Campylobacter jejuni* is the leading cause of bacterial diarrhoea worldwide, surpassing the occurrence of infections by other well-known enteric pathogens [1]. In the developed world, *C. jejuni* infection and colonization of the human gut, leading to gastroenteritis, most commonly occurs following the consumption of contaminated chicken meat [2]. Campylobacter remains the most prevalent bacterial cause of gastroenteritis in the EU [3–5] and recent studies from Ireland have shown the rate of contamination at multiple stages of meat processing remains high with up to 66 % of the caecal samples testing positive for *Campylobacter* [6, 7].

Once contaminated, the prolonged survival of *C. jejuni* on chicken meat within the supermarket is a major challenge associated with this pathogen. The survival of this pathogen in the aerobic, cold supermarket environment is surprising as it is characterized as microaerophilic and preferably grows at 37 °C or 42 °C, the temperature of the human and avian gut, respectively [8, 9]. However, several studies have reported examples of aerotolerance in fresh *Campylobacter* isolates [10–12].

Since the 1980s there has been a rise in the level of fluoroquinolone resistance in *C. jejuni* that has promoted this pathogen to a priority ‘high’ ranking from the WHO for research and development [13, 14]. Fluoroquinolone resistance is most often conferred through single nucleotide polymorphisms (SNPs) of the target of these antibiotics, DNA gyrase subunit A (GyrA) [15, 16], which in turn can lead to a change in DNA supercoiling, that has previously been linked to the regulation of virulence of *C. jejuni* [17, 18]. We recently reported that the selection for mutants in the laboratory strain NCTC11168 that are resistant to fluoroquinolones, results in clones that produce a greater amount of viable biofilm under aerobic conditions, show an increase in virulence and have a more relaxed DNA supercoiling profile in comparison to the wild-type [19]. Acquisition of fluoroquinolone resistance through mutations in *gyrA* has also been shown to increase fitness *in vivo* [20, 21].

Although many studies have documented the genetic diversity inherent in *Campylobacter* populations very little is known about any consequent phenotypic diversity and whether these phenotypes are selected at different stages of the infection cycle. The aim of this study is to phenotypically compare a panel of strains made up of isolates from different stages of the infection cycle, including fresh abattoir chicken isolates, supermarket chicken isolates and human clinical isolates. Specifically, we aim to identify whether phenotypes are being selected at different stages of the infection cycle and whether fluoroquinolone resistance is associated with the phenotypes of strains isolated from these environments.

## Methods

### Bacterial strains and growth conditions

*Campylobacter* isolates were isolated from supermarket chickens by swabbing from different sites on the surface of the whole chicken and growing on Mueller–Hinton (MH) plates containing Skirrow selective supplements for 5 days at 42 °C until single colonies could be confirmed as *Campylobacter* by PCR as outlined below. Isolates from chickens at the abattoir were sourced from Professor Paul Whyte at UCD and isolates from stool samples of *Campylobacter *-infected children were sourced from Dr Adele Habbington in Childrens Health Ireland Crumlin, Dublin, Ireland. Strains NCTC11168 and 81–176 were included to represent well characterized and highly passaged laboratory strains. All strains used in this study were stored at −80 °C in MH broth solution containing 20 % glycerol. When required strains were streaked from frozen stocks onto MH agar plates and incubated under microaerophilic conditions at 37 °C, generated using atmosphere generating gas packs (CampyGen – Oxoid) for 3 days. MH broth cultures were established from 3 day plate cultures (initial inoculum OD_600_ 0.02, 10 ml) and maintained under microaerophilic conditions, generated using CampyGen gas packs, at 37 °C or 42 °C with shaking at 200 r.p.m.

### Species confirmation by PCR

Genotypic confirmation to the species level for each isolate was carried out using primers designed to differentiate using the *ceuE* as previously described [22]. PCR reactions were prepared as per the protocol of the MyTaq DNA polymerase Red Mix. PCR template was prepared from fresh plate cultures as follows. A loop of bacteria was resuspended in 100 µl PBS and then incubated at 100 °C for 10 min. The samples were centrifuged at 13000 r.p.m. for 10 min and the resulting supernatant diluted 1 in 10 for the final PCR template. PCR conditions consisted of an initial heat shock of 95 °C 1 min, followed by 40 cycles of a denaturing step of 95 °C for 15 s, an annealing step of 50 °C for 15 s and an extension phase at 72 °C for 10 s. Gel electrophoresis was used to visualize PCR products to confirm species identity.

### Motility assay

Isolates were grown on MH agar plates from frozen stocks for 3 days at 37 °C before being inoculated into motility agar plates that were made from MH broth containing 0.3 % agar. Each motility plate was inoculated with a single isolate using a sterile 200 µl tip. Motility plates were incubated at 37 °C and 42 °C under microaerophilic conditions for 48 h and the resulting diameter of motility measured and expressed as a percentage of the diameter of the plate.

### Antibiotic susceptibility assay

To assess the susceptibility of the isolate panel to ciprofloxacin and gentamicin the agar dilution method was used. Strains were cultured on MH agar plates from frozen (−80 °C) stocks and then inoculated into MH broth and grown overnight. Overnight culture samples were then normalized to an OD_600_ of 0.1 and 10 µl of this suspension inoculated onto MH agar plates containing a range of concentrations of ciprofloxacin or gentamicin. Plates were incubated for 72 h, under microaerophilic conditions at 37 °C. Following this incubation, the growth of each isolate was recorded, allowing for each to be categorized as resistant or sensitive based on breakpoint values and the resistant strains were subsequently characterised as displaying low (no growth >0.5 µg ml^−1^), medium (low growth at 25 µg ml^−1^) or high (high growth at 25 µg ml^−1^) resistance.

### Culture and maintenance of HT29 cell cultures

HT29 cells were cultured in McCoys medium containing 10 % FBS and 1 % glutamine. Cell cultures were incubated at 37 °C in a humid environment of 5 % CO_2_ and 95 % air. Upon reaching 80–90 % confluency cells were trypsinized for 10–15 min to remove dead cells and debris and to detach cells from the petri dish. Trypsinized cells were centrifuged at 200 r.p.m. for 5 min and the pellet resuspended in fresh complete medium. Cells were counted using a haemocytometer and seeded at a density of 10^5^ ml^−1^.

### Invasion assay

Cells growing at 80–90 % confluency were trypsinized as described above and counted using a haemocytometer. For invasion assays HT29 cells were seeded onto 13 mm coverslips in 12-well plates at a concentration of 1×10^5^ cells ml^−1^ and incubated at 37 °C for 4–5 days. Once HT29 cells had reached 90–100 % confluency invasion assays were carried out. Bacterial cells were maintained as described above and on the day prior to invasion were inoculated in MH broth at an OD_600_ value of 0.02 and incubated overnight at 37 °C, shaking (200 r.p.m.), under microaerophilic conditions. Following overnight growth, bacterial cells were washed four times in PBS by centrifugation. Once washed cells were then normalised to an OD_600_ of 0.2 in complete media and placed over the HT29 monolayers in the 12-well plates and the plate was then incubated for 3 h under microaerophilic conditions at 37 °C. Following this incubation, wells were washed three times with PBS and the cells fixed for 15 min using 4 % paraformaldehyde. Cells were blocked using PBS supplemented with 10 % goat serum and 1 % BSA, for 1 h. Once blocking was complete, a polyclonal rabbit-anti-* C *. *jejuni* antibody was added at a concentration of 1 : 200 in PBS and incubated for 45 min. Cells were washed three times in PBS and the goat-anti-rabbit-Alexa488 antibody was added at a concentration of 1 : 400 in PBS and incubated for 30 min. Cells were washed three times in PBS and finally the nuclei of cells were stained using Hoechst33342 (1 : 5000) for 10 min and washed with PBS. Cells on coverslips were imaged using an Olympus BX51 fluorescent microscope and the images quantified and analysed within Image J. The number of bacteria that had adhered to or invaded the HT29 cells, was estimated by first counting the number of bacteria in a field of view and then the number of nuclei. The number of bacteria was then divided by the number of nuclei to calculate the cell-association level of each isolate.

### Biofilm formation assay, crystal-violet staining

To assess the ability of the isolates to form biofilm at 37 °C under microaerophilic and aerobic conditions a crystal-violet assay was carried out. Strains were first cultured on MH agar plates from frozen (−80 °C) stocks and then cultured in MH broth overnight. Overnight cultures were normalized to an OD_600_ of 0.05 and 200 µl were seeded in duplicate into 96-well plates along with a corresponding broth control. Plates were incubated for 72 h, under microaerophilic or aerobic conditions at 37 °C. Following this incubation, the biofilm formed was visualized using 1 % crystal-violet stain. First, the broth was removed and the wells washed three times with PBS and dried at 37 °C for 10 min. Then, 200 µl of 1 % crystal violet was then added to each of the wells for 20 min. The crystal violet was removed and the wells washed three times with PBS. Elution buffer was added to each of the wells and the OD_595_ value of each well recorded using a SpectraMax Plus 384 microplate reader.

### Assessment of growth under aerobic conditions

To assess the oxygen tolerance of the isolate panel strains were first cultured on MH agar plates from frozen (−80 °C) stocks for 2–3 days. From these plate cultures two sets of MH agar plates were streaked and the plates incubated under microaerophilic or aerobic conditions at 37 °C. The resulting growth for the aerobic plates was recorded as intolerance (no growth visible) or low or high tolerance.

### Statistical analysis

Statistically significant differences between the means (*n*=3, biological replicates) of isolate groups defined by isolate source or ciprofloxacin resistance level were determined using a *T*-test with Welches correction. Statistically significant differences were denoted as per the guidelines of GraphPad Prism version 9.2.0 (283) software (i.e. *P≤0.05, **P≤0.01, ***P≤0.001 and ****P≤0.0001).

## Results

### Confirmation of isolates as *C. jejuni* or *C. coli*

A panel of *Campylobacter* isolates from three distinct stages in the infection cycle was assembled to provide an overview of changes in the phenotypic profile of these *Campylobacter* populations. *Campylobacter* isolates from chickens freshly processed at the abattoir (16 isolates), supermarket chickens (17 isolates) and clinical isolates from patients with *Campylobacter* infection (16 isolates) were assessed by PCR of the *ceuE* gene (38) to confirm the species identity of each isolate (Table 1). All strains were confirmed as *C. jejuni* apart from two isolates from abattoir chicken and one clinical isolate, which was identified as *C. coli *.

**Table 1.** Isolate panel consisting of two well characterized laboratory strains (NCTC11168 and 81–176) and 49 fresh isolates. All isolates were confirmed as *C. jejuni* or *C. coli* by PCR using primers specific to the *ceuE* gene

Isolate source	* C. jejuni *	* C. coli *	Total
**Lab**	2	0	2
**Abattoir chicken (CC**)	14	2	16
**Supermarket chicken (S**)	17	0	17
**Human isolate (HI**)	15	1	16

### Growth and motility are influenced by isolate source

**Fig. 1.**
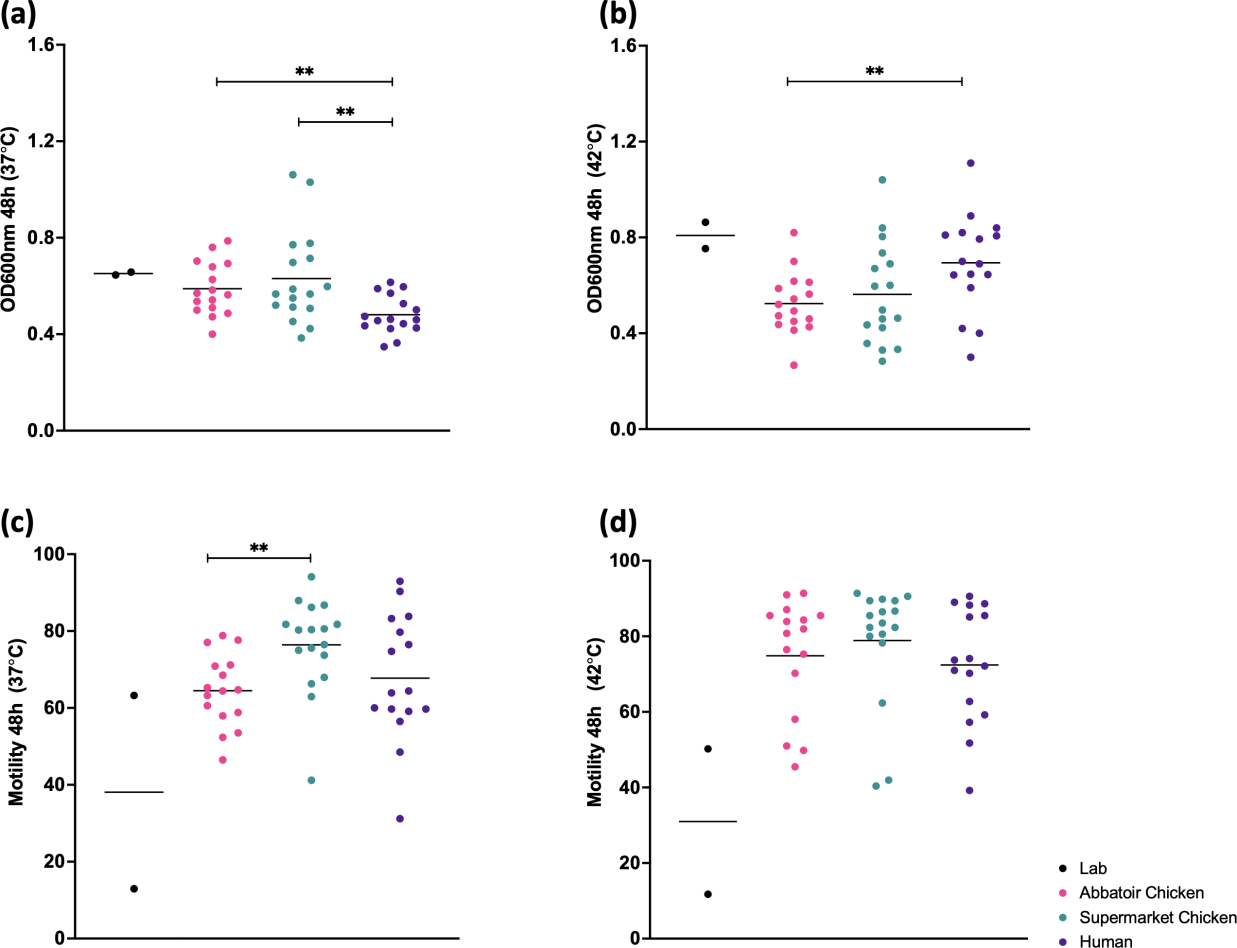
Growth (**a,b**) and motility (**c,d**) of isolates from different sources at 37 °C (**a,c**) and 42 °C (**b,d**) under microaerophilic conditions. Clinical isolates displayed significantly lower growth after 24 h when compared to isolates from abattoir (*P*=0.0115) or supermarket (*P*=0.0110) chickens at 37 °C but significantly higher growth than abattoir chicken isolates (*P*=0.0091) at 42 °C. Supermarket chicken isolates displayed the highest motility at 37 °C and were significantly more motile than abattoir chicken isolates (*P*=0.035).

The impact of the isolate source on the growth and motility of isolates was assessed. Growth in liquid culture of the isolate groups was measured as the OD_600_ values at 48 h, at 37 °C and 42 °C, following the incubation of an initial, normalized, inoculum of an OD_600_ of 0.02 (Fig. 1a, b). The clinical strains isolated from patient stool samples showed significantly lower growth at 37 °C and significantly higher growth at 42 °C than the abattoir or supermarket chicken isolates. After growth at 37 °C for 48 h, the highest motility was observed within the supermarket isolate group (Fig. 1c). At 42 °C (Fig. 1d) a similar pattern was observed but the difference was no longer statistically significant between the source groups.

### Clinical isolates display the lowest biofilm and highest cell-association capacity when compared with strains from abattoir and supermarket

**Fig. 2.**
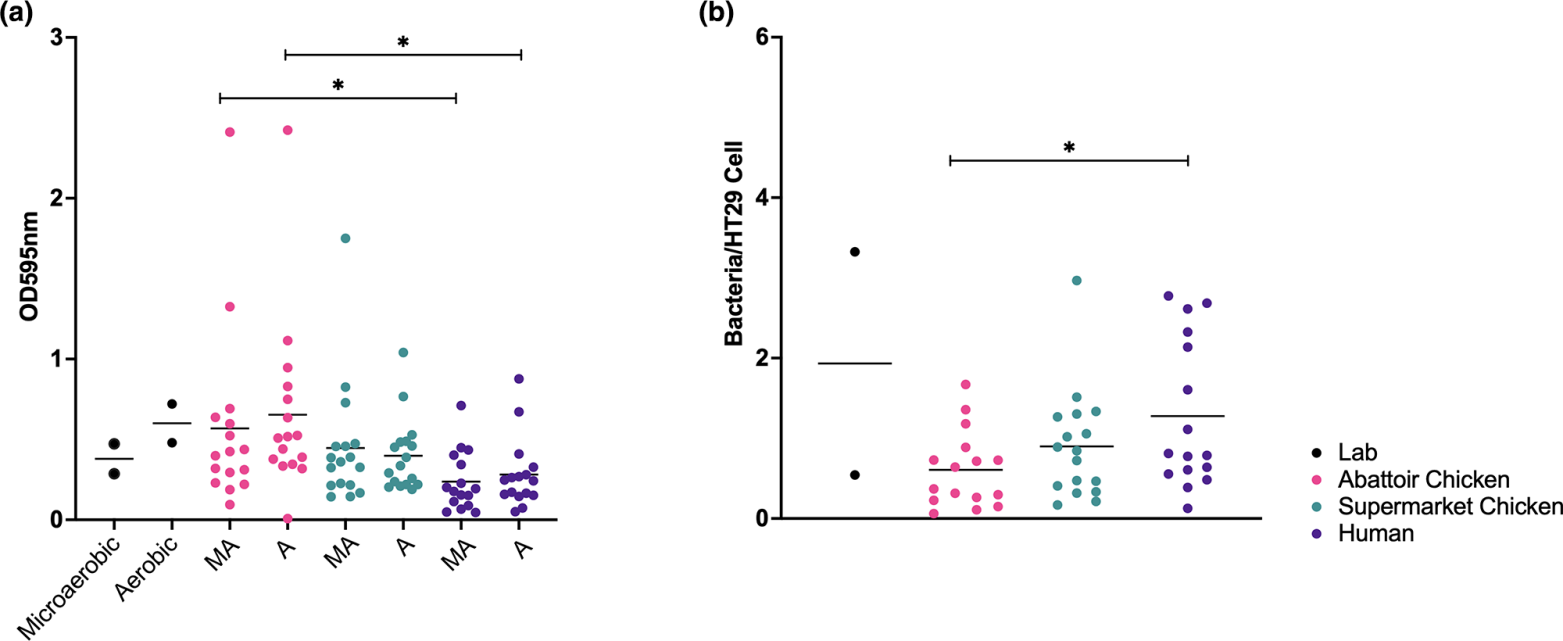
Biofilm (**a**) and cell-association density (**b**) of isolates from different sources. Biofilm formation under both microaerobic and aerobic conditions was highest in abattoir chickens, lower in isolates from supermarket chicken and lowest in the human clinical isolates. The difference between the biofilm formation of abattoir chickens and human clinical isolates was shown to be statistically significant under both microaerobic (*P*=0.04) and aerobic conditions (*P*=0.0196). Cell association revealed the opposite pattern with human clinical isolates revealed to show statistically significantly higher cell association to isolates from abattoir chicken (*P*=0.017).

To test whether isolate source could affect other phenotypes, the ability of the isolates to form biofilm (Fig. 2a) or their ability to associate with human epithelial cells (Fig. 2b) was assessed. Interestingly, the clinical isolates, on average, showed the lowest levels of biofilm formation along with the highest levels of epithelial cell association. These were followed by the supermarket isolates that showed a slightly higher average biofilm formation and a slightly lower level of cell association followed by the abattoir isolates, which had the lowest levels of cell association but conversely the highest levels of biofilm formation.

### Fluoroquinolone resistance is prevalent across isolate groups but is highest within the supermarket isolate group

To assess the fluoroquinolone sensitivity of the isolate panels the agar dilution method was used. Antibiotic plates containing a range of concentrations of ciprofloxacin were prepared along with plates containing gentamicin for comparison with a non-fluoroquinolone antibiotic. Fluoroquinolone resistance was found in all isolate groups except the two laboratory strains that were found to be sensitive to both antibiotics. In chicken and human isolates, 56 and 44 %, respectively, were found to be resistant to fluoroquinolone antibiotics (Table 2). The highest proportion of resistance, 94 %, was found within the supermarket isolate group.

**Table 2.** Comparison of antibiotic resistance in *Campylobacter* isolates from different sources. The highest prevalence of ciprofloxacin resistance was observed within the supermarket chicken isolate group (94 %) with a lower prevalence in the abattoir (56 %) and human (44 %) cohorts

Isolate source	Ciprofloxacin resistance %	Gentamicin resistance %
**Lab**	0 %	0 %
**Abattoir chicken**	56 %	0 %
**Supermarket chicken**	94 %	6 %
**Human isolate**	44 %	13 %

### Fluoroquinolone resistant and sensitive isolates show similar levels of growth and motility

**Fig. 3.**
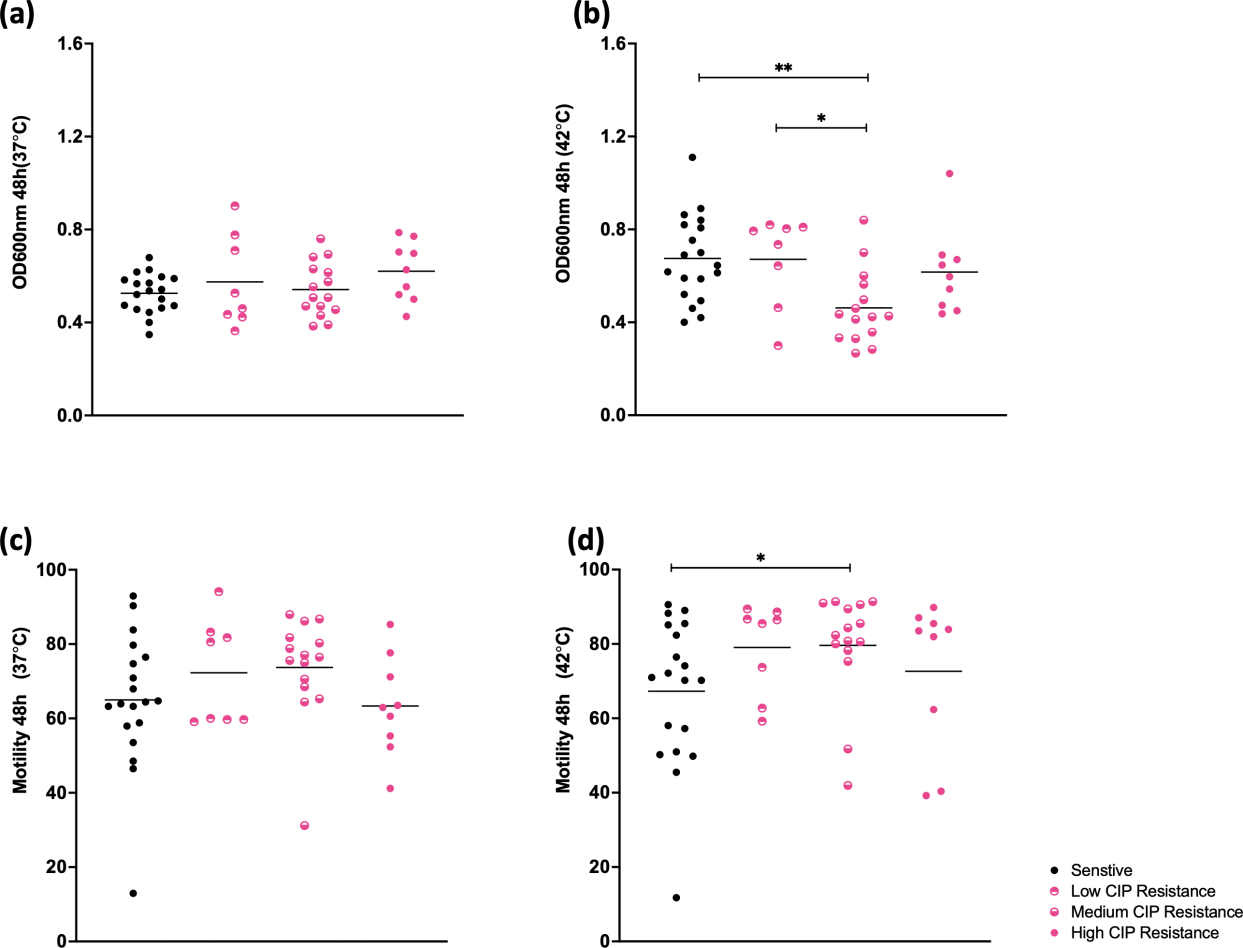
Growth (**a,b**) and motility (**c,d**) of isolates separated as being sensitive or displaying low, medium or high ciprofloxacin resistance. Very little difference was observed in growth of the different isolate groups at 37 °C although isolates displaying medium resistance to ciprofloxacin displayed a significantly lower growth after 48 h at 42 °C when compared to sensitive isolates (*P*=0.001) or those with low resistance (*P*=0.0217). Motility appeared to be increased in isolates displaying resistance to ciprofloxacin at both 37 and 42°C but this difference was only statistically significant at 42 °C between sensitive isolates and isolates with medium resistance (*P*=0.044).

Isolates were subsequently divided into those that were sensitive to ciprofloxacin (no growth at 0.5 µg ml^−1^), displayed low levels of resistance (no growth >0.5 µg ml^−1^), displayed medium levels of resistance (low growth at 25 µg ml^−1^) and those that displayed high levels of resistance (high growth at 25 µg ml^−1^). Overall, fluoroquinolone resistant and sensitive isolates displayed similar growth and motility levels in the absence of antibiotics at 37 and 42 °C (Fig. 3) at 48 h. Interestingly, the isolates characteriszed as having medium resistance to ciprofloxacin appeared to have significantly lower growth (medium CIP resistance Vs, sensitive *P*=0.001, low CIP resistance *P*=0.0217) and higher motility (*P*=0.044) than sensitive isolates at 42 °C but this difference was not apparent at 37 °C.

### No association observed between ciprofloxacin resistance and cell association but high levels of fluoroquinolone resistance are associated with high biofilm formation

**Fig. 4.**
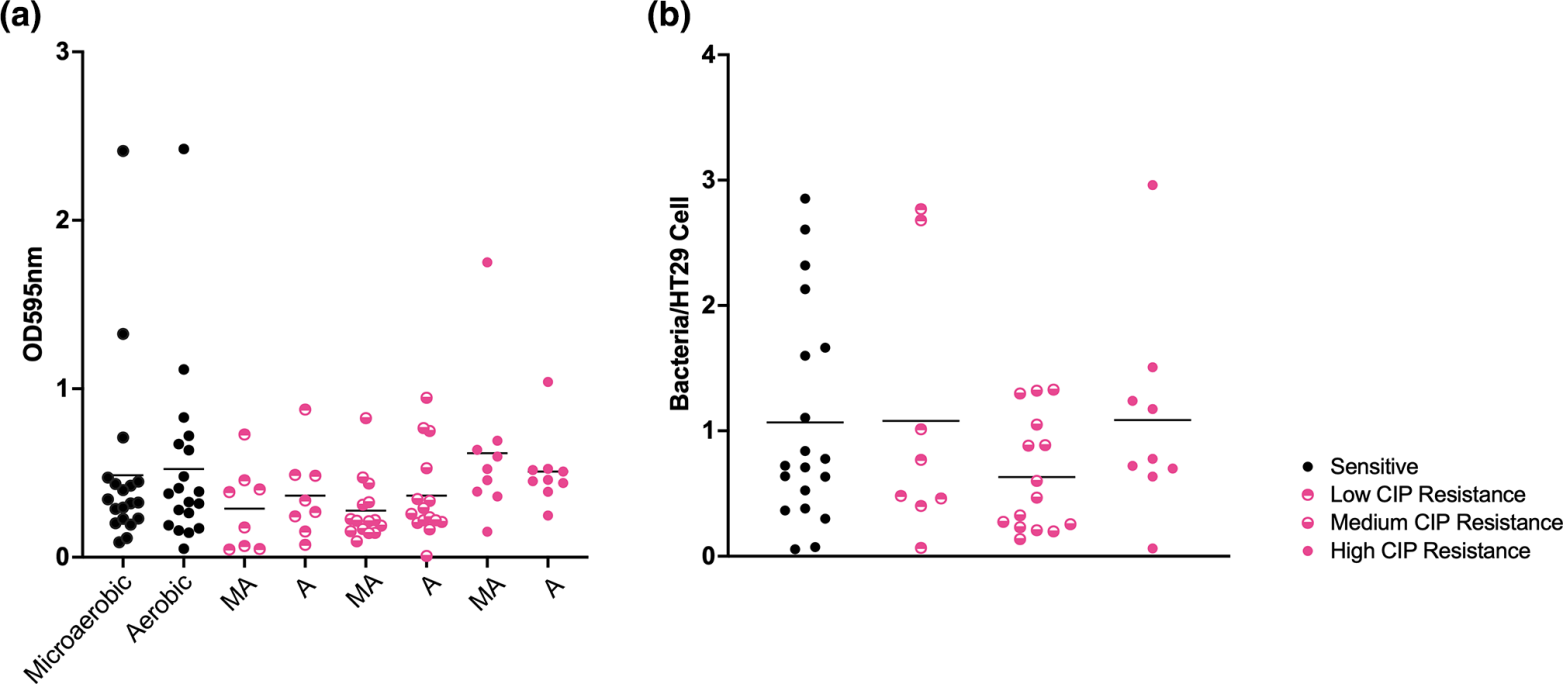
Biofilm (**a**) and cell-association density (**b**) of isolates separated as being sensitive or displaying low, medium or high ciprofloxacin resistance. Biofilm formation was similar in all groups although isolates with high ciprofloxacin resistance exhibited a higher average level of biofilm this was not statistically significant. When comparing cell-association density, no statistically significant differences were observed. Although isolates with medium ciprofloxacin resistance showed a lower level of cell association this was not statistically significant.

To test for an association between ciprofloxacin resistance and biofilm formation and cell association, the resistant isolates were again separated into groups by low, medium and high resistance to the antibiotic. Fluoroquinolone resistance had little effect on cell-association levels and although a lower level of cell association was seen in strains with medium resistance to the antibiotic this difference was not statistically significant (Fig. 4b). In the case of biofilm formation, the strains with highest resistance to ciprofloxacin displayed, on average, a high level of biofilm formation under both microaerobic and aerobic conditions (Fig. 4a).

### The ability of strains to grow under aerobic conditions is more prevalent among strains with a high resistance to ciprofloxacin

**Fig. 5.**
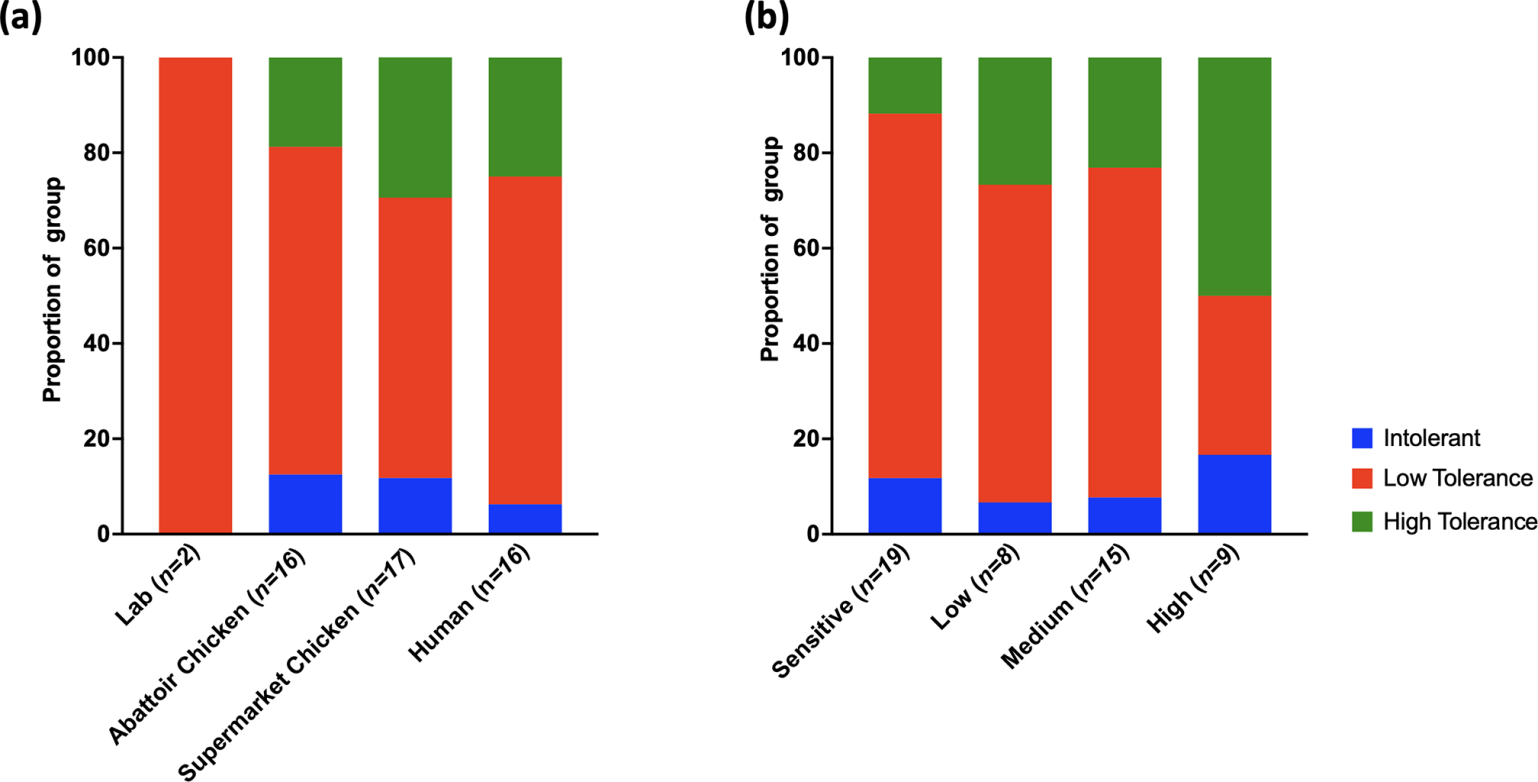
Assessment of the association between growth on plates under aerobic conditions and isolate source (**a**) and ciprofloxacin resistance (**b**). The highest tolerance of oxygen as evidenced by growth under aerobic conditions was observed in isolates from the supermarket environment and isolates with the highest level of resistance to ciprofloxacin were most likely to show a higher tolerance of oxygen.

The ability of the isolates to grow under atmospheric oxygen was also assessed using a simple streak plate assay. Visible growth following 48 h for each isolate was scored from intolerant to low or high tolerance depending on the level of growth observed on the plate. Isolates from all stages of the infection cycle were found to have a higher level of oxygen tolerance in comparison to the laboratory isolates with the highest proportion of isolates displaying high tolerance to oxygen being found within the supermarket group (Fig. 5a). Interestingly, ciprofloxacin resistant and sensitive isolates also differed in terms of their oxygen tolerance. A notable increased level of aerobic growth was observed in isolates that displayed a high level of ciprofloxacin resistance compared to isolates that were ciprofloxacin sensitive or had a lower level of resistance (Fig. 5b).

## Discussion

*C. jejuni,* the leading cause of bacterial diarrhoea worldwide has proven particularly difficult to combat due to our poor understanding of how it can colonize chickens in such high numbers, survive the aerobic conditions of the supermarket shelf and go on to cause gastrointestinal disease in humans. One of the biggest challenges is the inherent genetic variation observed between strains, which could lead to strains with very different phenotypes. This study compares freshly isolated strains from key stages of the infection cycle and compares them to each other and to two commonly used laboratory strains to reveal not only a large level of phenotypic diversity between strains but specific phenotypic traits enriched in strains from specific stages of the infection cycle.

Isolates from infected patients displayed the lowest level of growth at 37 °C and the highest level of growth at 42 °C. When comparing motility a high degree of variation was seen between isolates, which could reflect the fact that motility is regulated by phase variation and DNA supercoiling both of which vary from strain to strain. Interestingly the supermarket isolates displayed the highest average level of motility suggesting they may be primed for motility. When observing the change in phenotypic profile from abattoir to supermarket to human on average isolates tended to form lower levels of biofilm and higher levels of cell association. This suggests the possibility that strains expressing phenotypes useful for human colonization are being selected as they move through the infection cycle or are changing their phenotypes as they do so.

Fluoroquinolone resistance was observed across all cohorts but was most evident in the isolates from supermarket chicken. The high number of resistant supermarket isolates was particularly interesting as we have previously shown that some mutations that cause fluoroquinolone resistance are associated with changes in DNA supercoiling that can have a consequent effect on a variety of phenotypes including invasion, motility and, critically for this cohort, survival under aerobic conditions [19]. It was thus notable that isolates with the highest level of fluoroquinolone resistance did display a greater ability to tolerate growth in the presence of oxygen suggesting this phenotype may be more strongly associated with or influenced by fluoroquinolone resistance. However, further work is required to fully understand the observed association between aerobic tolerance and fluoroquinolone resistance.

The inherent diversity observed between isolates and the clear association between different phenotypes and different stages of the infection cycle is striking along with the observed associations between fluoroquinolone resistance in isolates from the supermarket and between high levels of fluoroquinolone resistance and the ability to tolerate growth in oxygen. Whole-genome analysis of larger cohorts of phenotypically characterized strains will be critical in future studies to confirm whether specific mutations are being selected at different stages of the infection cycle to affect these changes in phenotype.
